# Noninvasive positive pressure ventilation in the assessment of extrinsic tracheal stenosis

**DOI:** 10.1093/icvts/ivac044

**Published:** 2022-03-03

**Authors:** Alfonso Fiorelli, Roberta Fiorito, Gaetana Messina, Francesco Leone, Rosa Mirra, Maria Caterina Pace, Fausto Ferraro, Mario Caterina

**Affiliations:** Thoracic Surgery Unit, University of Campania “Luigi Vanvitelli”, Naples, Italy; Anaesthesia and Intensive Care Unit, University of Campania “Luigi Vanvitelli”, Naples, Italy; Anaesthesia and Intensive Care Unit, University of Campania “Luigi Vanvitelli”, Naples, Italy; Thoracic Surgery Unit, University of Campania “Luigi Vanvitelli”, Naples, Italy; Thoracic Surgery Unit, University of Campania “Luigi Vanvitelli”, Naples, Italy; Anaesthesia and Intensive Care Unit, University of Campania “Luigi Vanvitelli”, Naples, Italy; Anaesthesia and Intensive Care Unit, University of Campania “Luigi Vanvitelli”, Naples, Italy; Thoracic Surgery Unit, University of Campania “Luigi Vanvitelli”, Naples, Italy

**Keywords:** Airway stenosis, Positive pressure, Stent

## Abstract

In patients with extrinsic tracheal stenosis caused by a mediastinal mass, an airway stent is a palliative measure to relieve airway obstruction. However, the self-expanding force of the stent may be insufficient to force a rigid stenosis. Our goal was to report a simple strategy to indirectly estimate the rigidity of the stenosis and predict airway patency after inserting the stent. Before the procedure, the inspiratory and expiratory flows and their ratio were evaluated under spontaneous breathing and after positive pressure ventilation generated by a facial mask. In patients with stenosis successfully treated with a stent (n = 11), we found significant changes in expiratory (2.3 ± 0.7 vs 2.8 ± 0.7; p = 0.03) and inspiratory (1.5 ± 0.6 vs 2.5 ± 0.9; p = 0.001) flows and a reduction of their ratio (1.4 ± 0.3 vs 1.1 ± 0.2; p = 0.01) whereas no significant changes were observed in patients (n = 2) whose stent failed to force the stenosis. In these cases, a tracheostomy was performed to assure ventilation. Our simple strategy may help physicians predict airway patency after stenting or plan alternative treatments in patients with rigid stenosis difficult to force by stenting.

## INTRODUCTION

Airway stenting is a palliative measure to assure ventilation in inoperable patients with extrinsic tracheal compression caused by a mediastinal mass [1]. However, the self-expanding force of the stent may be insufficient to force a rigid stenosis. We reported a simple strategy using noninvasive positive pressure ventilation (NPPV) to estimate indirectly the rigidity of the stenosis and predict airway patency after stenting.

## METHOD

Before beginning the endoscopic treatment of extrinsic airway stenosis, a full-face mask was applied to the patient, who was lying in a supine position and connected to the ventilator. The peak expiratory flow (PEF), and the peak inspiratory flow (PIF) were then recorded under spontaneous ventilation and after administration of NPPV with the positive end-expiratory pressure set at 10 cm H_2_O [1, 2]. The PEF/PIF ratio was then calculated. The mean values of PEF and PIF and the PEF/PIF ratio before and after NPPV were statistically compared using the *t*-test, and a *P*-value < 0.05 was considered statistically significant (MedCalc statistical software; Version 12.3, Mariakerke, Belgium).

During spontaneous ventilation, extrinsic airway stenosis selectively impairs PIF rather than PEF as a result of negative intratracheal pressure generated during inspiration. This action elevates the PEF/PIF ratio. NPPV generates positive intratracheal pressures both in inspiration and in expiration, resulting in an improvement of respiratory loops that is more marked for PIF than for PEF [1, 2]. Thus, we expect a reduction in the PEF/PIF ratio after positive pressure in patients with no firm stenosis and no significant changes in those with firm stenosis.

A signed informed consent was obtained from all patients for this procedure, and they were aware that their data could be used anonymously for scientific purposes only.

## RESULTS

This procedure was applied in 13 consecutive patients undergoing covered self-expanded metallic stenting (SEMS) via rigid bronchoscopy for extrinsic compression of the trachea due to a large mediastinal mass (lymphoma *n* = 12 and thyroid carcinoma *n* = 1). All patients presented with severe dyspnoea and were unfit for surgical treatment.

**Figure 1:**
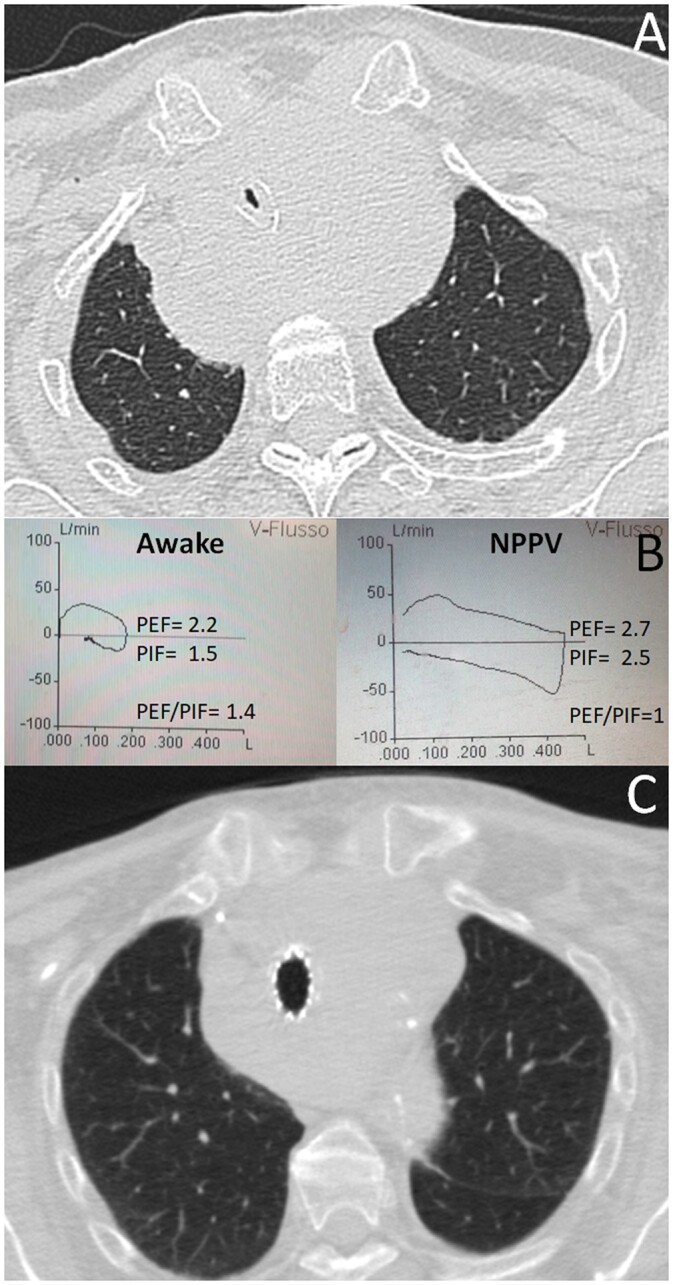
(**A**) A 79 -year-old woman with extrinsic trachea compression due to a lymphoma; (**B**) significant changes in the flow-volume loops after positive pressure predicted a not-firm stenosis; (**C**) the stenosis was successfully treated with a stent.

In 11 cases, the stent successfully reopened the airway without any complications. In 3 cases, the stent was removed 3.5 ± 0.7 months later due to reduction of airway stenosis after chemo- and radiotherapy. An improvement in the PEF (2.3 ± 0.7 vs 2.8 ± 0.7; *P* = 0.03) and in the PIF (1.5 ± 0.6 vs 2.5 ± 0.9; *P* = 0.001) and a reduction in the PEF/PIF ratio (1.4 ± 0.3 vs 1.1 ± 0.2; *P* = 0.01) were observed before and after NPPV. An example is shown in Fig. 1 and Video 1.

**Figure 2:**
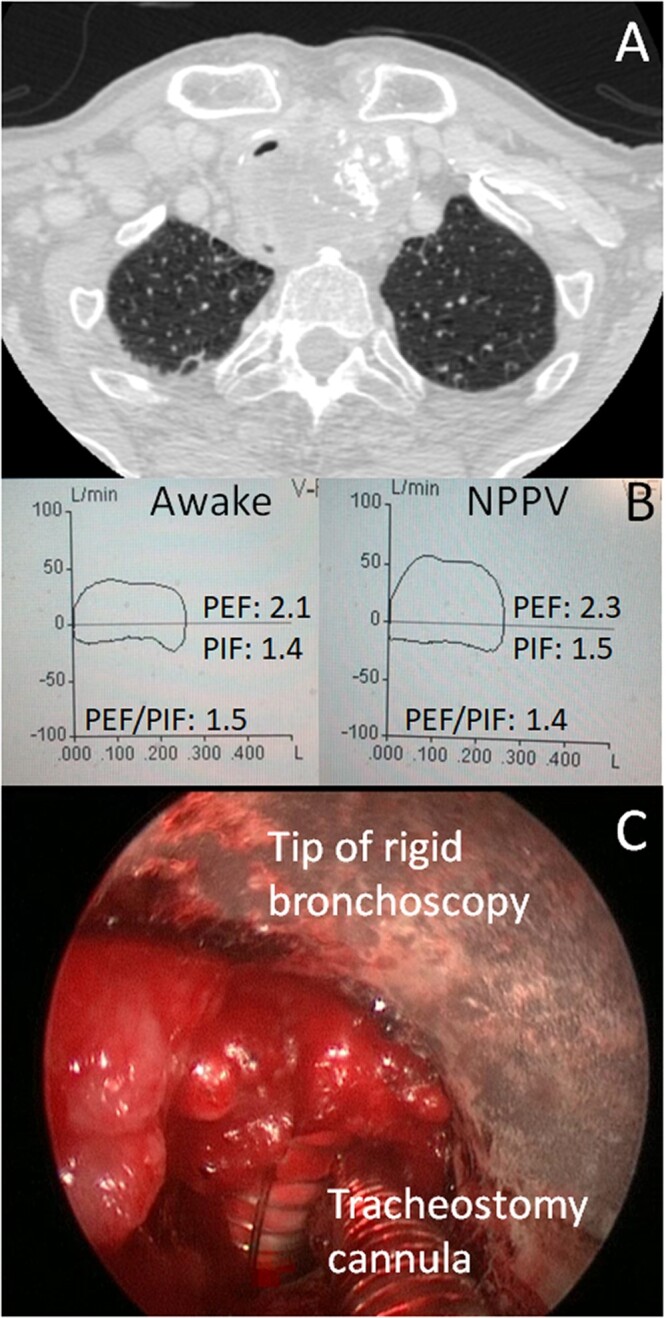
(**A**) A 67 -year-old man with extrinsic tracheal compression due to thyroid cancer; (**B**) the lack of significant changes in the flow-volume loops after positive pressure predicted a firm stenosis; (**C**) a percutaneous dilatational tracheostomy guided by rigid bronchoscopy was performed.

In 2 patients, the stent failed to treat the stenosis. A percutaneous dilatational tracheostomy (PDT) guided by rigid bronchoscopy in 1 case and a surgical tracheostomy in the other case were performed to assure ventilation. No significant changes in PEF (2.1 ± 0.7 vs 2.3 ± 0.8; *P* = 0.65), PIF (1.4 ± 0.4 vs 1.5 ± 0.3; *P* = 0.77) and the PEF/PIF ratio (1.5 ± 0.5 vs 1.4 ± 0.24; *P* = 0.49) were observed before and after NPPV. An example is shown in Fig. 2.

## DISCUSSION

Tracheal stenosis is a life-threatening condition caused by postintubation injury, an intraluminal tumour or extrinsic compression by a tumour. Endoscopy is a palliative treatment in inoperable patients. Generally, the resection of a stenotic scar and/or an intraluminal tumour restores airway patency, whereas in patients with extrinsic stenosis, airway stenting is indicated for re-opening the airway [1]. However, the success of this procedure depends on the characteristics of the stenosis. In patients with firm stenoses, the self-expanding force of the stent may be unable to force the stenosis, and physicians should be prepared to perform an emergency intubation or tracheostomy to prevent fatal airway obstruction. These procedures are extremely challenging, especially in emergencies due to the difficult airway and the presence of a mediastinal mass.

Thus, the preoperative assessment of the rigidity of the stenosis is mandatory to predict the success of airway stenting and to avoid this critical scenario. No strategies have been reported to evaluate this issue thus far. Spirometric variables have been used to detect the risk of critical airway collapse after induction of general anaesthesia [1, 3], but many patients with tracheal stenosis cannot perform spirometry due to severe respiratory distress, as was the case in all our patients. A computed tomography scan clearly defines the characteristics of the stenosis, but tracheal collapsibility during breathing is difficult to predict even with sophisticated imaging techniques [1].

Extrinsic compression of the trachea from goitres or anterior mediastinal masses may cause flow volume loop abnormalities, especially during the inspiratory phase due to negative intratracheal pressure generated during inspiration [1]. Nouraei *et al.* [2] used NPPV through a laryngeal airway mask for ventilating patients with laryngotracheal stenosis during induction of general anaesthesia. The NPPV generated positive intratracheal pressures in inspiration and in expiration, and the selective impairment to inspiratory airflow that was observed with the patient awake was minimized. Based on these findings, we argued that improvement in flows after positive pressure could identify those stenoses that were suitable for stenting, whereas no changes indicated a rigid stricture that could not be palliated with a stent. To test this hypothesis, the PEF/PIF ratio was compared before and after positive pressure. In line with the data of Nourei *et al.* [2], we found in patients successfully treated with a stent a significant improvement in flows that was more evident for inspiratory (PIF) than for expiratory (PEF) flow and a reduction of the PEF/PIF ratio compared to awake values. In contrast, no significant changes were found in 2 patients in whom the stent was unable to force the stenosis. In 1 of these patients, a PDT was performed by rigid bronchoscopy as previously reported by others [4–6], whereas the other patient underwent a surgical tracheostomy. Our results were in line with the experiences of other authors [7–9] who evaluated the changes in spirometric values in relation to the characteristics of a benign airway stenosis. They found that a fixed scar produced a constant degree of limited airflow, resulting in a similar flattening of the inspiratory and expiratory loop without a significant change in the forced expiratory flow at 50%/forced inspiratory flow at a 50% (FEF50%/FIF50%) ratio, which was similar to that of normal subjects. In contrast, flaccid stenosis presented a flattening at the inspiratory (extrathoracic stenosis) or expiratory phases (intrathoracic stenosis) with significant changes in the FEF50%/FIF50% ratio.

There were no specific contraindications for our strategy. During the procedure, the mask should be firmly secured to the patient’s face to prevent air leaks that could affect the measurements. Additionally, we performed the evaluations in patients lying in the supine position, because it reproduced the same clinical situation as that created during rigid bronchoscopy and stent deployment. However, the supine position could exacerbate the compression of the trachea by the mediastinal tumour, resulting in acute respiratory failure that may be managed by placing the patient in Fowler's position and applying NPPV. Recently, Saito *et al.* [10] used CT scans in combination with NPPV to predict the impact of positive pressure ventilation during general anaesthesia in a patient with tracheal stenosis caused by a mediastinal tumour and scheduled for airway stenting. This strategy allows evaluation in real time by a CT scan the changes in diameter of tracheal stenosis in response to positive pressure, but it requires the transfer of the patient to the radiology unit where is it is difficult to monitor the airway. In contrast, our method is performed at the bedside, which can turn out to be very useful especially in an emergency situation, when the CT scan is not readily available or cannot be performed to evaluate the patient’s clinical condition.

Obviously, our results should be considered with caution before drawing definitive conclusions because of the small sample size and the different characteristics of mediastinal masses. Furthermore, we used a covered metallic stent, because all patients had malignant stenoses. Thus, it remains unclear whether our results are also reproducible with silicone stents, which have a lower expansion force than metallic stents.

In conclusion, our strategy is simple, does not require specific equipment and is performed at the patient’s bedside, even in emergency situations. Significant changes in flows and in the PEF/PIF ratio after positive pressure could predict a not-rigid stenosis likely to be forced by stenting whereas no changes suggest a rigid stenosis. Thus, in these cases, physicians should plan alternative treatments to assure ventilation due to the risk of stent failure.

## Notes

This paper was presented as an oral presentation at the 29th Meeting of the European Society of Thoracic Surgeons 20–22 June 2021—Virtual Meeting (Best Mixed Abstracts I Session II; Sunday 20 June; 12.25; Abstract number 172)
